# Bilateral sensorineural hearing loss and cerebellar ataxia in the case of late stage Lyme disease

**DOI:** 10.5935/1808-8694.20120046

**Published:** 2015-10-20

**Authors:** Pierre Bertholon, Céline Cazorla, Anne Carricajo, Alexander Oletski, Bernard Laurent

**Affiliations:** ^1^ MD (Department of Otolaryngology, Head and Neck Surgery, Saint Etenne University Hospital); ^2^ MD (Department of Infectous Diseases); ^3^ MD (Laboratory of Bacteriology, Virology and Hygiene, Saint Etenne University Hospital); ^4^ MD (Department of Neurology, Saint Etenne University Hospital)

**Keywords:** cerebellar ataxia, hearing loss, bilateral, Lyme disease

## INTRODUCTION

Stage three or late Lyme disease is characterized by chronic neuroborreliosis^1^. We present a case of bilateral hearing loss due to late stage Lyme disease and discuss the interrelation of hearing loss regarding the stage of the disease.

## CASE REPORT

**Figure 1:**
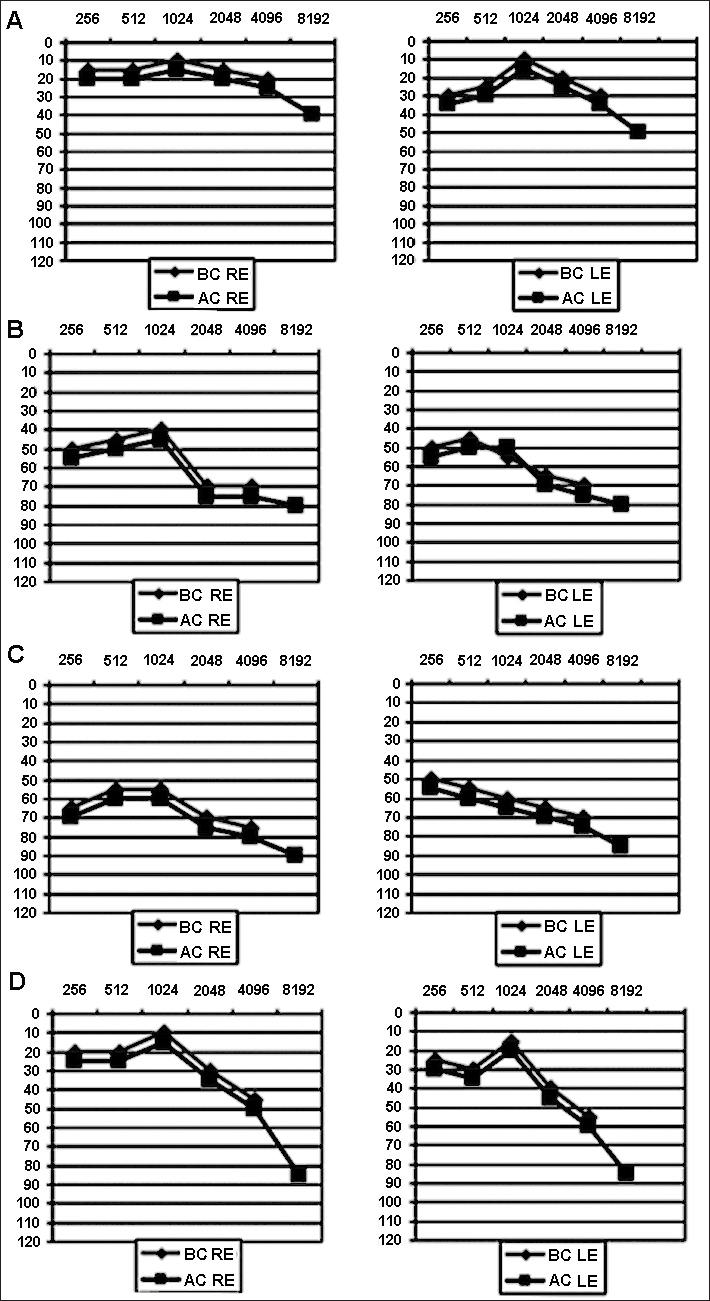
Pure tone audiometry. A: July 2, 2004; B: September 12, 2006; C: April 24, 2007; D: March 3, 2009. AC: Air Conduction; BC: Bone Conduction; RE: Right Ear; LE: Left Ear.

A 61-year-old woman was hospitalized on March, 2007 for a progressive bilateral hearing loss with a gait disorder. She had a history of migraine and depression. She also suffered from vertigo of unknown origin, which had been thoroughly investigated in 2004, with a near normal audiometry (Figure 1A). Her health insidiously deteriorated at the end of 2005, with an aggravation of her headache. Brain MRI scan was normal. In January 2006, she was depressed. She developed frequent vomiting in March 2006 and had fatigue.

There was no abnormality in serum inflammatory markers. In the summer 2006, her hearing progressively deteriorated bilaterally (Figure 1B). At the beginning of 2007, she complained of a permanent gait disorder due to a cerebellar syndrome. Pure tone audiometry showed an aggravation of the hearing loss (Figure 1C). Speech audiometry was in keeping with pure tone audiometry. Brain MRI scan was normal (March, 2007). A cerebrospinal fluid examination (April 2, 2007) showed a predominantly lymphocytic pleocytosis (344 white cells; 90% of lymphocytes) with elevated protein (2.7 g/l). Serology for Lyme disease was positive in plasma (VIDAS Lyme IgG and IgM) and in both plasma and cerebrospinal fluid (Western Blot).

Different serologies including syphilis, HIV, cytomegalovirus, listeria, brucellosis were negative. Results of all immunologic analyses were normal. Treatment was started on April 10, 2007 consisting of Ceftriaxone (8 weeks) followed by Doxycycline (3 weeks) with gradual improvement of gait and hearing. When reassessed in March 2009, the patient had returned to normal activities of daily living although still retained a slight dysequilibrium and hearing loss (Figure 1D).

## DISCUSSION

The patient, who had a previous and misleading history, developed atypical symptoms at the end of 2005 that can secondarily be related to Lyme disease due to the presence of a lymphocytic meningitis, the detection of high titer antibodies and the efficacy of antibiotic treatment.

The unique feature was the bilateral hearing loss which developed insidiously in the summer 2006. There are two cases of progressive bilateral sensorineural hearing loss due to late stage Lyme disease^2^, ^3^. In these two cases, the hearing loss had a long duration, was associated with neurological symptoms, and was successfully treated by antibiotic treatment. However, a hearing loss has been reported in 12 out of 48 patients suffering from late stage Lyme disease although this finding was not documented by audiometry^1^. Thus, we can conclude that a progressive bilateral hearing loss occurs in late stage Lyme disease^1^, ^2^, ^3^. This feature is different from a sudden unilateral hearing loss which is controversially linked to stage 2 Lyme disease with a positive serology ranging from 21%^4^ to 0%^5^. Serological positivity to Borrelia must be viewed with caution due to false positive results, and the clinical diagnosis of stage 2 Lyme disease was difficult to affirm as the sudden hearing loss was isolated (no associated neurological symptoms)^4^, ^5^, ^6^, the cerebrospinal fluid analysis was normal^6^, and antibiotic treatment had a debatable efficacy^4^, ^5^, ^6^.

## CLOSING REMARKS

A progressive bilateral sensorineural hearing loss associated with neurological disorders typically occurs in late stage Lyme disease (stage 3). This feature is different from hearing loss in stage 2 Lyme disease, which has essentially been reported to be unilateral and sudden, but for whom, probably due to the early stage of the disease, the causal relationship is more difficult to demonstrate.
